# Revisiting the remember–know task: Replications of Gardiner and Java (1990)

**DOI:** 10.3758/s13421-020-01073-x

**Published:** 2020-09-15

**Authors:** Julia M. Haaf, Stephen Rhodes, Moshe Naveh-Benjamin, Tony Sun, Hope K. Snyder, Jeffrey N. Rouder

**Affiliations:** 1 University of Amsterdam, Amsterdam, Netherlands; 2 Rotman Research Institute, Baycrest, North York, Canada; 3 University of Missouri, Columbia, MO USA; 4 University of California, Irvine, CA USA

**Keywords:** Recognition memory, Implicit memory, Replication

## Abstract

One of the most evidential behavioral results for two memory processes comes from Gardiner and Java (*Memory & Cognition, 18*, 23–30 1990). Participants provided more “remember” than “know” responses for old words but more know than remember responses for old nonwords. Moreover, there was no effect of word/nonword status for new items. The combination of a crossover interaction for old items with an invariance for new items provides strong evidence for two distinct processes while ruling out criteria or bias explanations. Here, we report a modern replication of this study. In three experiments, (Experiments 1, 2, and 4) with larger numbers of items and participants, we were unable to replicate the crossover. Instead, our data are more consistent with a single-process account. In a fourth experiment (Experiment 3), we were able to replicate Gardiner and Java’s baseline results with a sure–unsure paradigm supporting a single-process explanation. It seems that Gardiner and Java’s remarkable crossover result is not replicable.

One major feature in the modern study of memory is a healthy respect for the distinction between different mnemonic processes. One impactful distinction is that between conscious recollection and familiarity-based automatic activation (Atkinson & Juola, 1973; Jacoby, 1991; Mandler, 1980; Yonelinas, 2002). This distinction forms the basis of dual-process theory, and is influential in the neurobiology of memory (Squire, 1994; Vilberg & Rugg, 2008), in understanding cognitive aging (Jennings and Jacoby 1993, 1997; Prull, Dawes, Martin, Rosenberg, & Light, 2006), and in memory pathology research (Yonelinas, Kroll, Dobbins, Lazzara, & Knight, 1998).

Dual-process theory is a polarizing topic in memory research. Proponents of dual-process theory cite several pillars of behavioral support, including the shape of the receiver operating characteristic (Yonelinas, 1999), the presence of double-dissociations in explicit and implicit recognition tasks (Schacter & Tulving, 1994), speed-effects associated with different processes (Besson, Ceccaldi, Didic, & Barbeau, 2012; McElree, Dolan, & Jacoby, 1999), and the selective influence of critical manipulations in the remember–know–new paradigm (Tulving, 1985). Skeptics, on the other hand, have provided what we call demonstrations of doubt (e.g., Dougal and Rotello 2007; Dunn 2004; Mulligan and Hirshman 1995). The prevailing argument of the skeptics is that it is possible to account for the above phenomena with a single process rather than with two distinct processes. For example, asymmetries in ROC signatures may arise from two separate processes, but may reflect specific configurations within a single-process account (Province & Rouder, 2012). Likewise, double dissociations across tasks may also be accounted for by a single monotonic performance curve that reflects the operations of a single process (Dunn, 2008). Yet, many of these demonstrations of doubt are contestations of what counts as evidence more than firm findings. Consequently, whether individual researchers find single- or dual-process theories more convincing seems to reflect that individual’s training more than any specific finding.

One pillar of support that we find especially convincing is certain selective influence results within the remember–know–new paradigm. In the remember–know–new paradigm (Tulving, 1985), participants are first given a study list, and they are subsequently presented with a test list consisting of previously studied (old) and new items. Participants judge whether each test item is new or old. If they judge the item as old, then they further judge whether they *remembered* the item or *knew* it. According to proponents, the endorsement of a *remember* response indicates a conscious, recollective recall, and the endorsement of a *know* response indicates automatic activation based on familiarity.

The remember–know task may be combined with experimental manipulations to validate the claim that it measures distinct memory processes. Gardiner (1988), for example, used a levels-of-processing manipulation with the critical hypothesis that deeply processed items are more likely to be consciously recollected. Table 1 shows the results from Gardiner (1988, Experiment 1). Indeed, only *remember* responses are affected by the processing depth manipulation.

**Table 1:** Response proportions from Gardiner (1988, Exp. 1)

	Response		
	Remember	Know	New
Condition			
Deep	0.65	0.17	0.18
Shallow	0.35	0.17	0.48
Lure	0.05	0.07	0.88

Even though the above results are impressive, Donaldson (1996) and Dunn (2004) provide an important critique. Accordingly, the remember–know judgment cannot be considered a direct measure of memory processes without accounting for the influence of decision processes. An alternative to dual-process accounts is a single-process signal-detection account where *remember* and *know* responses reflect different criteria on a single latent mnemonic strength. A manipulation at study may affect decision criteria perhaps as much as the underlying memory strength (Hirshman and Master, 1997). Indeed, Dunn (2004) shows how the large corpus of remember–know results like those in Table 1 may be accommodated by a single-process signal-detection model.

There is, however, a class of remember–know results that seems immune to the Donaldson–Dunn critique. Consider the experiments from Gardiner and Java (1990), who had participants study words and word-like nonwords. At test, participants judged four types of items: old words, old nonwords, new words, and new nonwords. The key here is the inclusion of two types of lure: new words and new nonwords, which allows for the isolation of criterial effects. Both single- and dual-process theories may account for an increase in *remember* responses for old words compared to old nonwords. If this increase is due to criterial differences between words and nonwords, as stipulated by the single-process account, we would expect a corresponding increase in *remember* responses for new words relative to new nonwords. If this increase reflects enhanced recollection of old words, then there should be no difference in *remember* responses for new items. Note that for this paradigm there are clear predictions that may be assessed without the need for a formal process model.

The results of Gardiner and Java (1990), shown in Table 2, are stunning. For old items, there is a perfect crossover with a greater proportion of *remember* responses to old words and a greater proportion of *know* responses to old nonwords. For new items, response proportions are invariant to lexical status. This lack of effect implies that lexical status does not affect decision criteria. The strength of these results is the simultaneous demonstration of a perfect crossover in one condition with a perfect invariance in another. No process model is needed to interpret the data pattern. In our view, these results are perhaps the strongest of all remember–know results that we know of because they implicate two mnemonic processes while ruling out one.

Others have tried to reproduce Gardiner and Java’s key findings. Rajaram, Hamilton, and Bolton, (2002) report two experiments using the same general procedure as Gardiner and Java but a shorter retention interval of 15 min. In both experiments, they find the same item type (word, nonword) by rating (remember, know) interaction for old items. However, the equivalent analysis for new items (i.e., false-alarms) is not reported. In both cases, there appears to be a know-response bias for nonwords. Geraci, McCabe, & Guillory (2009, Experiment 1) report very similar results to Rajaram et al., (2002) and, in addition, report analyses of hits minus false-alarms in which the item type by rating interaction is not significant. They attribute this result to the high proportion of know responses to nonwords. Thus, while these findings have been treated as replications of Gardiner and Java’s central results, we find that they are quite ambiguous. A replication study showing the exact pattern of simultaneous crossover and invariance is warranted.

**Table 2:** Response proportions from Gardiner and Java (1990, Exp. 2)

	Response			
	Remember	Know	New	Scaled difference
Condition				
Old word	0.28 (0.26)	0.16 (0.2)	0.56 (0.55)	0.12
Old nonword	0.19 (0.19)	0.3 (0.3)	0.51 (0.51)	− 0.11
New word	0.04 (0.05)	0.11 (0.09)	0.85 (0.86)	− 0.07
New nonword	0.03 (0.03)	0.12 (0.12)	0.85 (0.85)	− 0.09

*Note.* Predictions from a single-process model are provided in parentheses

It appears that both proponents of dual-process accounts and single-process accounts have focused on the interpretation of the cross-over interaction for old items ignoring the invariance for new items. Dunn (2004), for example, fit a single-process model to the data where strength and criterion parameters are allowed to vary between words and nonwords. The predictions from Dunn’s model are shown in parentheses in Table 2. The absolute deviations from the observed data seem small, and this may be why Dunn did not interpret them as meaningful. Yet, the relative size and direction of the misses is clearly important: If we focus on old words, we see that the model only predicts half the effect (.12 vs. .06), and if we focus on the observed equality between new words and new nonwords, we see the model introduces a .04 effect, which is quite sizable for such small proportions. The direction and sign of these misses are the tell-tale sign of two processes. We may be the first to interpret the simultaneous crossover and invariance as so impactful.

Because Gardiner and Java (1990) results implicate the dual-process account at the expense of the single-process account, they serve as an appropriate target of replication. We performed a preregistered replication study across two different labs.^1^ The replication attempts spanned the labs of Rouder and Naveh-Benjamin, who have somewhat opposing views on the usefulness of the distinction between recollection and familiarity. In previous publications, Naveh-Benjamin and colleagues have leveraged the explanatory power of this distinction (e.g., Naveh-Benjamin et al. 2009; Old and Naveh-Benjamin 2008) while Rouder and colleagues have been skeptical (e.g., Pratte & Rouder, 2011, 2012).

## Statistical models for data analysis

The striking elements of Gardiner and Java (1990) results are the perfect crossover for old items in conjunction with an invariance for new items. While it is clear that this data pattern supports a dual-process interpretation, it remains unclear in general which possible data patterns would contradict this interpretation. In our view, identifying these patterns *before* data collection is key for a replication study. Therefore, before we collected any data, we proposed and preregistered the following models and analyses.

Gardiner and Java conducted a 2 × 2 ANOVA for old items where they treated lexical status and remember/know judgment as factors. The result was a significant interaction and no significant main effects. The authors interpreted this interaction as the critical piece of support theory. They then ran a separate 2 × 2 ANOVA for new items resulting in a non-significant interaction. They more or less disregarded this analysis. There are several flaws with this approach: 1. Remember–know judgments are not a factor. Hence, as explained below, the interpretation of the interaction is compromised. 2. Using separate tests to assess what happened for new and old items is unprincipled. Instead, a joint analysis of all key data patterns is warranted. 3. How test results correspond to theories is post hoc. For example, a significant interaction for old items could be due to the perfect crossover interaction observed by Gardiner and Java, or it could be a result of a completely different data pattern. Therefore, the test of interaction only becomes meaningful in combination with an inspection of the corresponding data plot. The theoretically predicted data pattern is much more precise than the test for *any* interaction pattern that was conducted. To correct these flaws, we decided to assess evidence for dual-process theory or single-process theory in a Bayesian model comparison framework. The key is that the models are specified before data collection.

Gardiner and Java imply two hypotheses. Their first hypothesis, denoted *H*_1_, is that *remember* responses would be more prevalent for old words than old nonwords. Their second hypothesis, denoted *H*_2_, is that *know* responses would be more prevalent for old nonwords than for old words. Though not explicitly hypothesized, Gardiner and Java tested the lack of effect of lexical status for new items. We call this null hypothesis *H*_3_.

Unfortunately, *H*_1_ and *H*_2_ are not independent: Any endorsement of a *remember* response necessarily implies a lack of endorsement of a *know* response leading to a negative correlation between *remember* and *know* response rates. The correlation is highest if one conditions on the number of *new* responses. If the *remember* response frequency is high, the *know* response frequency automatically has to be low. It is therefore not surprising that Gardiner and Java found a crossover interaction in their analysis. They treated *remember* and *know* responses as independent and overinterpreted the negative correlation pattern for old items.

One way to address this dependency is to model a composite measure rather than two independent measures: We simply take the difference of *remember* and *know* response frequencies which implicitly accounts for the negative relationship. Here, we provide a set of statistical models on this difference that specify the above hypotheses.

We start with the following notation for data. Let *r*_*ij*_, *k*_*ij*_, and *n*_*ij*_ denote the number of *remember*, *know* and *new* responses, respectively, for the *i* th participant and *j* th condition, *i* = 1,…,*I* and *j* = 1, 2, 3, 4, where the four conditions in order are: presentation of old words, old nonwords, new words, and new nonwords. Hence *r*_*ij*_ + *k*_*ij*_ + *n*_*ij*_ sums to the number of tested items for the *i* th participant in the *j* th condition. We model a single measure, the *scaled difference score*, denoted as *Y*_*ij*_, and define it as the scaled difference between *remember* and *know* responses:

1 $$  Y_{ij}=(r_{ij} - k_{ij})/(r_{ij} + k_{ij} + n_{ij}). $$

The value of the scaled difference is between -1 and 1, and it is negative when *know* responses are preferred over *remember* responses, and positive when *remember* responses are preferred over *know* responses. The last column in Table 2 provides scaled differences per condition for the response proportions in Gardiner and Java’s Experiment 2. What is critical here is both the sign of these scaled differences as well as the comparison across old and new items. First note that for old words the sign is positive for words and negative for nonwords indicating greater endorsement of *remember* for words (*H*_1_) and *know* for nonwords (*H*_2_). Moreover, note that the values of the scaled differences are about the same for new words and new nonwords. Certainly, no direction would be truly preferred for new items, but the critical point is the equality for new items (*H*_3_).

To test patterns of data, we develop a set of statistical models on the scaled differences, *Y*_*ij*_, that incorporate the critical patterns or their negation. The most general model, the *unconstrained model*, is

$$ {\mathcal M}_{u}: \quad Y_{ij}\sim\text{Normal}(\mu_{j},\sigma^{2}), $$

where *μ*_*j*_ is the true mean scaled difference for the *j* th condition, and *σ*^2^ is the common variance. The model that instantiates *H*_1_, *H*_2_ and *H*_3_ simultaneously obeys the following restrictions:

$$ \begin{aligned} {\mathcal M}_{*}: \quad \mu_{1} &> \mu_{2},\\ \mu_{3} &= \mu_{4}. \end{aligned} $$

The inequality constraint corresponds to the higher prevalence of *remember* responses for old words than to old nonwords. The equality corresponds to the lack of effect of lexical status for new items. This model may be compared to the unconstrained model that does not impose any ordering restrictions on the collection of *μ*_*j*_. In addition to the unconstrained model, we propose the following alternatives to competitively test model _∗_ against. The first model _1_ captures the case that the lexical status has no effect on the scaled difference for old or new items:

$$ {\mathcal M}_{1}: \quad \mu_{1} = \mu_{2},  \mu_{3}=\mu_{4}. $$

The second model _2_ captures the case that words, regardless of being old or new, enhance *remember* responses over *know* responses:

$$ {\mathcal M}_{2}: \quad \mu_{1} > \mu_{2},  \mu_{3} > \mu_{4}. $$

The third model _3_ captures the opposite case that nonwords, regardless of being old or new, enhance *remember* responses over *know* responses.

$$ {\mathcal M}_{3}: \quad \mu_{1} < \mu_{2},  \mu_{3} < \mu_{4}. $$

We follow Haaf, Klaassen, and Rouder, 2019 and Rouder, Morey, Speckman, and Province, (2012) for prior settings for *μ*_*j*_ and *σ*^2^, using a *g*-prior approach. The critical setting here is the scale on *g*, and we used a default setting of *r* = 2/2. With this setting, model comparison with Bayes factors is straight-forward using analytic solutions (Rouder et al., 2012) and the encompassing approach (Klugkist, Laudy, & Hoijtink, 2005). For the analysis, we used the BayesFactor package in R (Morey & Rouder, 2015). ded at https://github.com/PerceptionAndCognitionLab/rm-gardiner-java.

## Experiment 1

The goal of Experiment 1 was to closely replicate Gardiner and Java’s Experiment 2. Even so, we decided to improve the experimental methods in four ways outlined subsequently.

### Methods

In their Experiment 2, Gardiner and Java (1990) showed 20 participants 15 words and 15 nonwords on handwritten cards, sequentially, for 2 s each. Then, after a 24-h delay, participants were given a recognition test. Sixty items, again handwritten, were presented on a single piece of paper. These 60 consisted of 15 old words, 15 old nonwords, 15 new words, and 15 new nonwords. Participants were instructed to circle old words and then write “R” or “K” next to the item to indicate whether the old-item response reflect recollecting or knowing.

Here are the ways our experiment differed from Gardiner and Java. 1. To the best of our knowledge, at the time of designing this experiment, the original materials were not available (we were not able to contact the original authors). We therefore used different words and nonwords that were constructed following Gardiner and Java’s generation rules. 2. Instead of a 24-h retention interval, we used a 10-min retention interval filled with a distractor task. The reason for this change is as follows: Gardiner and Java explicitly justify their 24-h interval as a means of lowering performance to avoid ceiling effects (p. 24).

We decided that the better way to lower performance was to ask participants to remember more items. With more items, the statistical properties of the experiment increase and the experimenter has greater resolution to detect differences if they exist and greater confidence in null results otherwise. Moreover, asking participants to return is inconvenient and may result in the loss of some participants, introducing a new bias into the sample. Hence, using a 10-min delay with more items—in our case we doubled the number of to-be-remembered and to-be-judged items—is a preferred approach on all accounts to avoiding ceiling effects. 3. We increased the number of participants from 20 to 52 and doubled the number of items at study and at test. This increase of the number of observations results in a much better resolution of the data. 4. We used computer-presented items rather than handwritten ones. Both study and test were performed in a sequential manner rather than simultaneously.

#### Participants and design

Experiment 1 was conducted at the Perception and Cognition Lab at the University of Missouri. We initially planned to recruit 50 undergraduate students. In total, 53 undergraduates were recruited at the University of Missouri and participated for partial course credit. One participant was excluded from analysis due to overall performance below chance (accurate response in less than 50% of the trials). The study has a 2 (words vs. nonwords) x 2 (old vs. new items) repeated-measures factorial design, resulting in a total of 53 × 2 × 2 × 30 = 6360 collected observations.

#### Material

Criteria for material selection were taken from Gardiner and Java’s Experiment 2. Sixty high familiarity concrete nouns with one syllable and four letters were taken from the MRC psycholinguistics database (Coltheart, 1981). Sixty pronounceable nonwords with four letters and two to four phonemes were selected from the ARC nonword database (Rastle, Harrington, & Coltheart, 2002). The words and nonwords used in this study are shown in Appendix A. In the original study, the authors formed two fixed study sets of 15 words and 15 nonwords and randomly selected one of the lists for each participant. In our study, 30 words and 30 nonwords were chosen at random to form the study set for each participant. Items were presented at the center of the screen in the Lucida Console font with a height of 2^∘^ of visual angle at an approximate viewing distance of 50 cm. At test, 60 words and 60 nonwords were sequentially presented in a random order. Items were shown in the center of the screen together with two buttons (either labeled *OLD* and *NEW* or *R* and *K*, see below) that they could click on to respond. The buttons are circular with a radius of 2^∘^ and are presented 5^∘^ below and 5^∘^ to the left and right of the center of the screen.

#### Procedure

During the study phase, participants studied 60 items (30 words and 30 nonwords) in a randomly determined order. Each item appeared on the screen for 2 s (as in the original study) followed by a 0.5-s inter-stimulus interval. The test phase followed after a 10-min retention interval. During the retention interval, participants were given a “spot-the-difference” task to complete before moving on to the recognition test. For this task, participants were asked to compare two pictures with small changes between them and circle these changes. Afterwards, participants were given instructions for the recognition test phase. The instructions were presented on several screens, and are provided in Appendix B. After the instructions were given on the screen, the experimenter gave a few every-day examples of when *remember* and *know* responses may be appropriate.

This approach was also used by Gardiner and Java (1990), but the exact examples from the original study could not be employed as they were not reported. During the recognition test, participants were presented with items one at a time and characterized each item as *old* or *new* using the mouse to click on the corresponding button on the screen. Following an *old* response, participants then made an additional remember–know judgment by using the mouse to click on buttons labeled *R* (for *remember*) or *K* (for *know*).

### Results

Data were *born open* (Rouder, 2016), that is, they were uploaded to a public repository nightly during data collection, and are available https://github.com/PerceptionCognitionLab/data1/tree/master/repGardinerJava/RKN_replication/RKN_exp1. Details about the analysis code are provided in Appendix C. Average response proportions are shown in Table 3. Average accuracy on the old/new task was between 61% and 65% in all four conditions. This accuracy value is just a tad lower than the average accuracy, 66%, in Gardiner and Java’s Experiment 2. All-in-all, our 10-min retention period coupled with a doubling of items resulted in an overall performance level that was comparable to that from Gardiner and Java.

**Table 3:** Response proportions for replication studies

	Response			
	Remember/Sure	Know/Unsure	New	Scaled difference
Experiment 1				
Old words	0.35	0.30	0.35	0.05
Old nonwords	0.36	0.28	0.37	0.08
New words	0.20	0.18	0.61	0.02
New nonwords	0.20	0.15	0.64	0.05
Experiment 2				
Old words	0.35	0.31	0.35	0.04
Old nonwords	0.32	0.32	0.36	0.00
New words	0.08	0.23	0.69	-0.16
New nonwords	0.07	0.15	0.78	-0.09
Experiment 3				
Old words	0.46	0.19	0.35	0.28
Old nonwords	0.45	0.19	0.35	0.26
New words	0.18	0.16	0.66	0.01
New nonwords	0.13	0.17	0.70	-0.04
Experiment 4				
Old words	0.35	0.29	0.35	0.06
Old nonwords	0.42	0.26	0.32	0.15
New words	0.10	0.16	0.74	-0.07
New nonwords	0.13	0.17	0.70	-0.04

*Note.* Response proportions are for remember–know–new responses for Experiments 1, 2, and 4, and for sure–unsure–new responses for Experiment 3. The last column provides the scaled difference values per condition as specified in Equation 1

#### Descriptive analysis

Participants in our study displayed far less bias than those in Gardiner and Java’s. In our experiment, hit rates (0.63) and correct-rejection rates (0.64) are about the same in value indicating no particular bias to say old or new. This relative lack of bias contrasts to extreme bias in Gardiner and Java. In their experiments, hit rates were low (0.47) while correct-rejection rates were high (0.85).

**Fig. 1:**
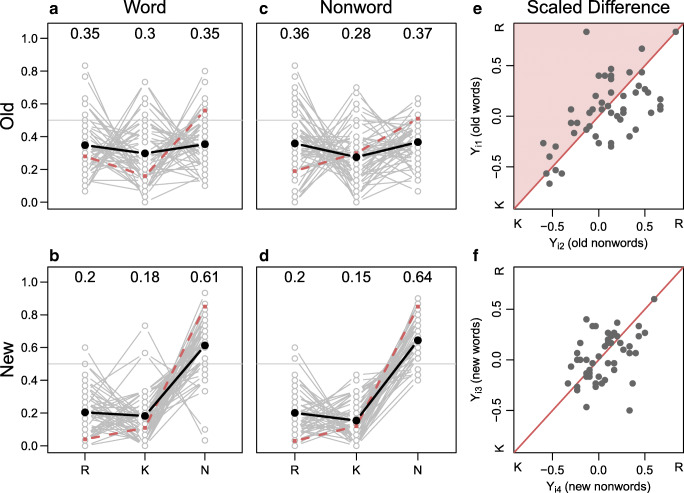
Results from Experiment 1. The *dark lines* shows average response rates for all participants; *dashed lines* show average response rates from Gardiner and Java (1990) Experiment 2. Critically, there is no interaction between item type (i.e., word vs. nonword) and preferred response category (i.e., remember vs. know) for the replication data. The *right two panels* show the modeled scaled difference scores for nonwords relative to words. According to dual-process theory, the scaled difference scores should be above the diagonal for old items as indicated by the shaded area, and on or close to the diagonal for new items

To assess the data pattern critical for the replication, we focus on proportions of *remember* and *know* responses as shown in Fig. 1. The black lines in panels A–D show average response proportions. The two left panels show response proportions to old and new words, and the two middle panels show response proportions to old and new nonwords. The original results by Gardiner and Java (1990) is shown by the dashed line. The critical comparison is between the left and middle panels of each row. The expected data pattern for a successful replication of Gardiner and Java (1990) would show the following two signatures: 1. A marked difference between the left and middle panels of the top row. In particular, recollection responses should be higher for old words than old nonwords and the reverse for know responses. 2. No differences between the bottom left and bottom middle panels; that is, there should not be an effect of lexical status for new items. We did not observe the first signature. Panel A appears to be the same as panel C. The invariance between the left and middle panels indicates that there is no effect of lexical status on responses for old or new items. Nonwords seemingly act like words.

It may seem surprising that there is no effect of lexical status. However, note that Gardiner and Java (1990) also failed to find a main effect of lexical status (see Table 2). Instead, their analysis showed a perfect crossover interaction of lexical status and response category (*remember* vs. *know*). However, in the current study, there is no apparent interaction, let alone the stunning crossover.

On an average level, there is no differential preference for either *remember* or *know* responses across old and new items. Yet, individuals’ response proportions vary drastically as shown by the grey lines in Fig. 1. Some participants almost exclusively use *remember* responses to classify old items while others almost exclusively use *know* responses to classify old items. This variability of preferences may have various explanations, one of them being that participants are not able to consistently classify their mnemonic experience as *remember* or *know*. We return to this issue when discussing Experiment 2, which aimed to better instruct participants on the criteria for remember and know responses.

#### Model-based analysis

To quantify the evidence for or against the replication, we use the model-based approach explained previously. For this approach, we calculate scaled differences *Y*_*ij*_ for each individual and condition. As a reminder, these scaled differences can be interpreted as the bias for *remember* responses compared to *know* responses. On the group level, we expected a positive scaled difference for old words, a negative scaled difference for old nonwords, and similar scaled differences for new words and nonwords. Table 3 shows the average scaled differences for the conditions. All are positive, and the contrasts between the scaled differences for old items and new items are about the same.

Figure 1 panels E–F show the individual scaled differences for the four item types (old words and old nonwords in panel E; new words and new nonwords in panel F). If an individual experienced differing processing for words and nonwords as proposed in Gardiner and Java (1990), we should observe points above the diagonal line in panel E. Yet, the scaled differences across conditions are on both sides of the diagonal, and they are highly correlated suggesting a more global bias to one of the two response options.

The data in Fig. 1e and f are submitted to the model analysis, and the replication model, _∗_, is compared to alternative accounts using Bayes factor model comparison. The preferred model is Model _1_, the model representing a straight-forward single-process criterion shift account. According to the model, proportions of *remember* and *know* responses are about the same for words and nonwords. Model _1_ is preferred over the replication model _∗_ by 12.33-to-1. The second-best performing model is model _3_ with a Bayes factor of 4.71-to-1 in favor of the winning model. The least preferred model is model _2_ with a Bayes factor of 22.60-to-1 in favor of the winning model.

In summary, we were not able to replicate the data pattern in Gardiner and Java’s Experiment 2 (1990). Instead, the Bayesian analysis yields evidence for the alternative model _1_, capturing the case that the lexical status (nonword vs. word) has no effect on the scaled difference of *remember* and *know* responses for both old and new items.

### Discussion

There are similarities and differences between our results and Gardiner and Java (1990). Although our participants have the same overall accuracy as Gardiner and Java, they differ in bias. Our participants displayed no preference for old or new responses while Gardiner and Java’s were heavily biased toward new responses. Two procedural differences possibly may have contributed to this difference: 1. we used a sequential presentation at test reducing dependencies among responses to different items; and 2. we used more items with a shortened retention interval to control overall accuracy. We think the lack of bias is an improvement from a psychometric point-of-view and have no desire to change our procedure to reintroduce such bias. We provide context for interpreting these procedural differences in the General discussion.

There are two smaller concerns with Experiment 1. First, the overall accuracy is somewhat low. From a statistical point-of-view, it would be more desirable to have accuracy closer to .75. To raise the level of accuracy in Experiment 2, we slightly reduced the number of studied items from 60 to 50. Consequently, the number of to-be-judged items at test lowered from 120 to 100. Second, in Experiment 1 only the on-screen instructions were standardized. Participants read these with an experimenter, and then the experimenter provided a few every-day examples. This aspect of the procedure followed Gardiner and Java. However, we did not record the examples, and we cannot guarantee that different participants did receive the same examples with the same wording from different experimenters. In Experiment 2, we standardized our examples as well as instructions.

## Experiment 2

### Methods

#### Participants

Experiment 2 was conducted at the Memory and Cognitive Aging Lab at the University of Missouri. For the preregistration, we planned to collect at least 30 participants and up to 50 participants. We decided that Spring break 2018 would be our cutoff: If we collected more than 30 participants by then we would stop data collection; if not, we would continue until the end of the semester. Since all the confirmatory analyses are conducted in a Bayesian framework, optional stopping or data peaking was not considered problematic (Rouder, 2014). In total, 51 undergraduates were recruited at the University of Missouri and participated for partial course credit. The experiment has the same design as Experiment 1, resulting in a total of 51 × 2 × 2 × 25 = 5100 collected observations.

#### Material

Fifty words and nonwords were selected from Experiment 1, and the presentation parameters were identical. The selected words and nonwords are indicated in Appendix A.

#### Procedure

The general procedure was identical to that used in Experiment 1 with the following changes. Participants studied 50 items (25 words, 25 nonwords) in a random order and were tested on 100 items (50 old, 50 new). A major change was in the instructions presented prior to the recognition phase. We felt, following interaction with participants in Experiment 1, that the phrasing of the written instructions reported by Gardiner and Java could be improved. These experiments were reported almost 30 years ago and were conducted on a UK sample. We attempted to make the remember/ know distinction clearer for our younger, US educated participants. The instructions are provided in Appendix B.

### Results

**Fig. 2:**
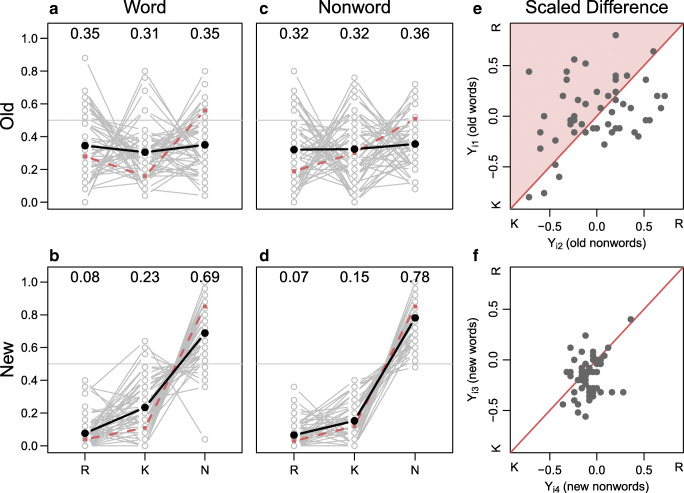
Results from Experiment 2. The *dark lines* shows average response rates for all participants; *dashed lines* show average response rates from Gardiner and Java (1990) Experiment 2. Critically, there is no interaction between item type (i.e., word vs. nonword) and preferred response category (i.e., remember vs. know) for the replication data. The *right two panels* show the modeled scaled difference scores for nonwords relative to words. According to dual-process theory, the scaled difference scores should be above the diagonal for old items as indicated by the *shaded area*, and on or close to the diagonal for new items

Data were made public after data collection and are available at https://github.com/PerceptionCognitionLab/data0/tree/master/rm-gardiner-java. Average response proportions are shown in Table 3. On average, participants performed better for new items with average accuracies of 69% and 78% for new word and new non-word, respectively. For old items, average accuracies remained similar to the levels in Experiment 1 with accuracies of 64% and 63%. Individuals’ response proportions are shown in Fig. 2.

#### Descriptive analysis

Once again, the critical comparison is the comparison of panel A to panel C and panel B to panel D in Fig. 2. This comparison yields almost no differences between the relative proportions of *remember* and *know* as a function of lexicality for either old items (top row) or new items (bottom row). Again, there is no sign of the prominent crossover interaction of the original study. Additionally, we again find notable individual differences in the preference of either *remember* or *know* responses.

#### Model-based analysis

Table 3 shows the average scaled differences for the four item types (old words, old nonwords, new words, and new nonwords). The scaled difference for old words is small and positive indicating more *remember* responses; the scaled difference for old nonwords is zero indicating no preference between *remember* and *know* responses; and the scaled differences for new words and nonwords are negative indicating a preference for *know* responses. The pattern critically deviates from the original Gardiner and Java pattern for old nonwords. Here, no preference for *know* responses is found.

Figure 2 panels E–F show the individual scaled differences for the four item types (old words and old nonwords in panel E; new words and new nonwords in panel F). In panel E, the scaled differences are on both sides of the diagonal. In both panels, the correlations are relatively large and positive suggesting a more global bias to one of the two response options.

In Bayes factor model comparison Model _1_, the model representing a straight-forward single-process criterion shift account, is preferred. According to the model, proportions of *remember* and *know* responses are about the same for words and nonwords. Model _1_ is preferred over the replication model _∗_, which is the second-best performing model. The Bayes factor between _1_ and _∗_ is 2.18-to-1 in favor of _1_. The least preferred model is model _2_ with a Bayes factor of 17.90-to-1 in favor of the winning model.

In summary, the main feature of Experiment 2 is a failure to replicate the stunning data pattern of Gardiner and Java’s Experiment 2. In fact, we replicated our Experiment 1 finding in that there is no effect of lexicality on recognition memory. We again found strong individual preferences to either *remember* or *know* responses. This finding may suggest that participants were not able to distinguish between these two distinct mnemonic experiences. To address this concern, we attempted to replicate Gardiner and Java’s Experiment 3, where participants are instructed to state the certainty of their *old*-response instead of *remember/know*.

## Experiment 3 – sure vs. unsure instructions

Although our focus has been on Gardiner and Java’s Experiment 2, these authors ran an additional experiment (Experiment 3), to show that the crossover interaction was unique to the remember–know instructions, and, by extension, that remember and know can be interpreted as processes distinct from levels of confidence. In our Experiment 3, we aimed at replicating (Gardiner & Java, 1990) Experiment 3.

### Methods

In their Experiment 3, Gardiner and Java (1990) simply replaced *remember* with *sure* and *know* with *unsure* response options. In line with their expectation they found that, for both words and nonwords, participants responded *sure* more than *unsure* to old items, whereas for new words and nonwords *unsure* was selected more than *sure*. There were no effects of lexicality.

In our Experiment 3, we attempt to replicate Gardiner and Java’s Experiment 3 as a demonstration of calibration. If we replicate Experiment 3 of Gardiner and Java (1990) using similar experimental procedures to those in our Experiments 1 and 2, then we have higher confidence that our failure to replicate the more theoretically contentious findings of Gardiner and Java’s Experiment 2 is not due to procedural differences. We preregistered and conducted Experiment 3 at the same time as Experiment 2 and without knowing the results of Experiment 2.

#### Participants

Experiment 3 was conducted at the Perception and Cognition Lab at the University of Missouri. For the preregistration, we stated the same decision rule as for Experiment 2. In total, 51 undergraduates were recruited at the University of Missouri and participated for partial course credit. The experiment has the same design as the previous experiments, resulting in a total of 51 × 2 × 2 × 25 = 5100 collected observations.

#### Material and procedure

The same material as in Experiment 2 was used. The procedure was identical to Experiment 2 with two exceptions. First, participants received different instructions for the test phase guiding them on how to navigate sure/unsure responses. The instructions are provided in Appendix B. After the instructions, participants entered the test phase similar to Experiment 1 and 2. Participants were again presented with items one at a time and characterized each item as *old* or *new* using the mouse to click on the corresponding button on the screen. Following an *old* response participants then made a sure–unsure judgment instead of a remember–know judgment by clicking on buttons labeled *S* (for *sure*) or *U* (for *unsure*).

### Results

**Fig. 3:**
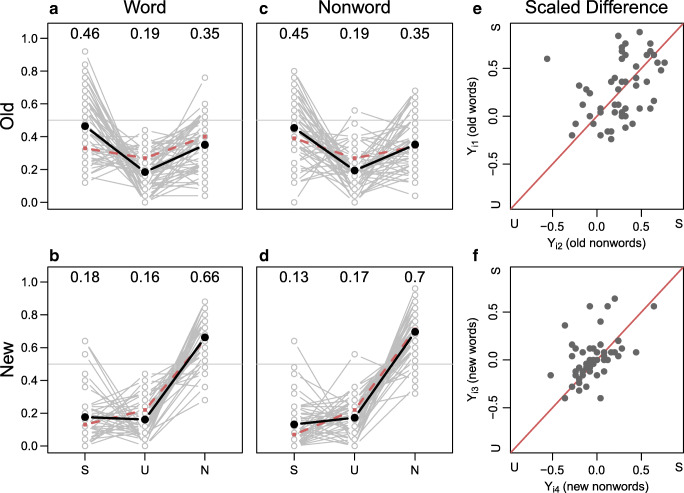
Results from Experiment 3. The *dark lines* show average response rates for all participants; *dashed lines* show average response rates from Gardiner and Java (1990) Experiment 3. The replication and original results are very similar. According to Gardiner and Java, the scaled differences shown in panels E and F should be on or close to the diagonal lines as no effect of lexicality is expected

Data were *born open* and are available at https://github.com/PerceptionCognitionLab/data1/tree/master/repGardinerJava/exp2/RKN_replication/RKN_exp2/SU. Average response proportions are shown in Table 3. On average, participants performed similarly for new and old items with average accuracies between 65% and 68%. On an individual level, accuracy varied between 24% and 96% when evaluated per condition. Individuals’ response proportions are shown in Fig. 3.

#### Descriptive analysis

The pattern of response proportions is fairly similar to the ones from Experiments 1 and 2 with the exception that there was a clear preference of *sure* responses over *unsure* responses for old items. In fact, the pattern of responses appears highly similar to Gardiner and Java’s Experiment 3 as shown by the dashed lines in Fig. 3. On an individual level, the majority of participants showed the response preferences for *sure* responses for old items, but there was no clear difference of preference between words and nonwords. For new items, *sure* and *unsure* responses were equally likely, again across words and nonwords.

#### Model-based analysis

Table 3 shows the average scaled differences for the four item types (old words, old nonwords, new words, and new nonwords). The scaled differences for old words and nonwords are large and positive indicating more *sure* responses than *unsure*. The scaled difference for new words is small positive, and for new nonwords is negative indicating a preference for *unsure* responses. This result corresponds to Gardiner and Java’s results for their Experiment 3.

Figure 3 panels E–F show the individual scaled differences for the four item types (old words and old nonwords in panel E; new words and new nonwords in panel F). As in the previous experiments, there is no sign for different response biases for words and nonwords. The positive correlation in the two graphs shows that individuals who prefer *sure* responses for words tend to also prefer *sure* responses for nonwords; participants who prefer *unsure* responses for words tend to also prefer *unsure* responses for nonwords. This pattern is in line with the original results in Gardiner and Java’s Experiment 3.

Bayes factor model comparison again shows a preference for model _1_, the model representing a straight-forward single-process criterion shift account. Model _1_ is the replication model for Gardiner and Java’s Experiment 3, and it is preferred over model _∗_, which is the second-best performing model. The Bayes factor between _1_ and _∗_ is 4.69-to-1 in favor of _1_. The least preferred model is model _3_ with a Bayes factor of 113.69-to-1 in favor of the winning model.

## Experiment 4

In our previous experiments, we attempted to instruct participants in a similar manner to Gardiner and Java, with Experiment 2 following stricter protocol than Experiment 1. However, since Gardiner and Java (1990) there has been growing acknowledgement of the importance of participant instruction in the R/K task (Geraci et al. 2009; Rotello, Macmillan, Reeder, & Wong, 2005), although a consensus on the most appropriate instructions has not been reached (Migo, Mayes, & Montaldi, 2012). Most relevant here is the work of Geraci et al., (2009), who report two experiments inspired by Gardiner and Java (1990). As noted in the Introduction, Geraci et al., (2009) found an interaction effect of lexical status for old items in their first experiment (but not the necessary same pattern for new items). In their first experiment, Geraci et al., (2009) used the instructions reported by Rajaram (1993), which were closely modeled after those proposed by Gardiner (1988). In their second experiment, they used different instructions (from Yonelinas, 2001) and did not find the same interaction. The explanation provided was that the Rajaram (1993) instructions do a better job of distinguishing “remembering” and “knowing” from confidence (e.g., by stating that knowing can be highly confident), whereas the Yonelinas (2001) instructions may conflate the two.

Given the seemingly crucial nature of R/K instructions, we conducted a final experiment in which the instructions were the same that were used in Experiment 1 of Geraci et al. (2009 see their Appendix A), with the exception of one important change. In the instructions of Geraci et al., (2009) the term “item” is used when discussing know responses, whereas “word” is explicitly used when discussing remember responses. We avoided this conflation in our instructions, using “item” in both cases. The exact instructions used can be found in Appendix B.

### Methods

#### Participants

Experiment 4 was conducted at the Memory and Cognitive Aging Lab at the University of Missouri. For the preregistration, we planned to at least collect 50 participants. Data collection was interrupted due to the current coronavirus crisis, but the total of 51 recruited undergraduates (University of Missouri) just exceeded the criterion. The experiment has the same design as Experiments 1 and 2, resulting in a total of 51 × 2 × 2 × 30 = 6120 collected observations.

#### Material

Sixty words (concrete nouns) and 60 non-words, with half of each from the original Gardiner and Java (1990) set and the other half from the additional stimuli created by Rajaram et al. (2002; and used by Geraci et al., 2009). All stimuli are made up of four letters. The stimuli are provided in Appendix A.

#### Procedure

The general procedure was identical to that used in Experiments 1 and 2. The only changes were the stimuli used and the presented instructions in the recognition phase. The full instructions are provided in Appendix B. The crucial change to the instructions is more emphasis on the notion that the experience of *knowing* is not equivalent to *unsure*. The examples provided in the instructions highlight this distinction:

If someone asks for your name, you would typically respond in the ’know’ sense, without becoming consciously aware of anything about a particular event or experience. However, when asked the last movie you saw, you would typically respond in the ’remember’ sense, that is, becoming consciously aware again of some aspects of the experience of seeing the movie.

### Results

**Fig. 4:**
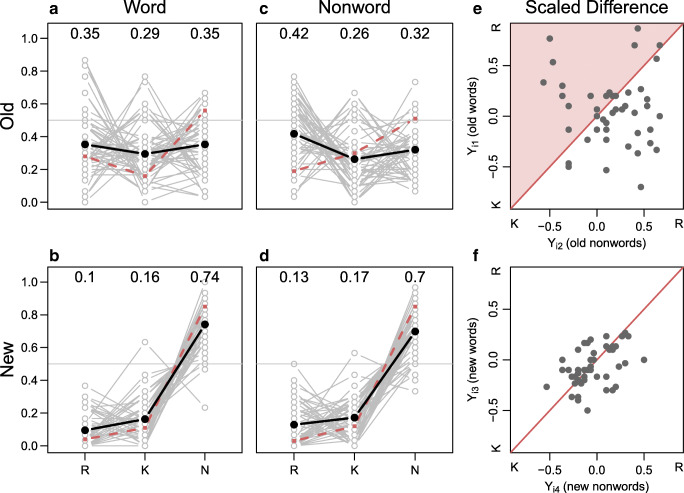
Results from Experiment 4. The *dark lines* shows average response rates for all participants; *dashed lines* show average response rates from Gardiner and Java (1990) Experiment 2. Critically, there is no interaction between item type (i.e., word vs. nonword) and preferred response category (i.e., remember vs. know) for the replication data. The *right two panels* show the modeled scaled difference scores for nonwords relative to words. According to dual-process theory, the scaled difference scores should be above the diagonal for old items as indicated by the shaded area, and on or close to the diagonal for new items

Data were made public after data collection and are available at https://github.com/PerceptionCognitionLab/data0/tree/master/rm-gardiner-java. Average response proportions are shown in Table 3. On average, participants performed better for new items with average accuracies of 74% and 70% for new words and new non-words, respectively. For old items, average accuracies were 65% and 68% for old words and non-words, respectively. Individuals’ response proportions are shown in Fig. 4.

#### Descriptive analysis

Once again, the critical comparison is the comparison of panel A to panel C and panel B to panel D in Fig. 4. This comparison yields almost no differences between the relative proportions of *remember* and *know* as a function of lexicality for either old items (top row) or new items (bottom row). There is seemingly a small *remember* response bias for non-words across old and new items when compared to words. But as in Experiments 1 and 2, there is no sign of the prominent crossover interaction of the original study. Additionally, we again find notable individual differences in the preference of either *remember* or *know* responses.

#### Model-based analysis

Table 3 shows the average scaled differences for the four item types (old words, old nonwords, new words, and new nonwords). The scaled difference for old words is small and positive indicating more *remember* responses; the scaled difference for old nonwords is a bit larger indicating some preference for *remember* responses over *know* responses; and the scaled differences for new words and nonwords are negative indicating a preference for *know* responses. The pattern critically deviates from the original Gardiner and Java pattern for old nonwords. Instead of the original preference for *know* responses we find a preference for *remember* responses. This difference is the same for new and old items indicating a response bias rather than differences in memory processing.

Figure 4 panels E–F show the individual scaled differences for the four item types (old words and old nonwords in panel E; new words and new nonwords in panel F). In panel E, the scaled differences are quite spread out and on both sides of the diagonal.

In Bayes factor model comparison Model _1_, the model representing a straight-forward single-process criterion shift account, is preferred. According to the model, proportions of *remember* and *know* responses are about the same for words and nonwords. Model _1_ is preferred over the replication model _∗_, which is the second-best performing model. The Bayes factor between _1_ and _∗_ is 12.33-to-1 in favor of _1_. The least preferred model is model _2_ with a Bayes factor of 71.42-to-1 in favor of the winning model.

In summary, Experiment 4 is our third attempt to replicate the stunning data pattern of Gardiner and Java’s Experiment 2—and we again fail to do so. Instead, our results are again close to the findings from our Experiments 1 and 2. The only deviation is that we find a small response bias toward *remember* responses for non-words. This deviation, however, is very much in line with single-process accounts of recognition memory. We again found strong individual preferences to either *remember* or *know* responses. This finding may suggest that, even with a third set of instructions, participants formed different impressions of what they were supposed to indicate, or simply were not able to distinguish between these two distinct mnemonic experiences.

## Additional analyses

In Experiment 1, 2, and 4, we attempted to replicate (Gardiner & Java, 1990) Experiment 2. We did not observe their stunning data pattern and show in model comparison that the statistical model in line single-process accounts of the remember/know paradigm was preferred over the model in line with dual-process accounts. Here, we provide additional analyses to put our results in context. First, we show that the replication was unsuccessful even if Gardiner and Java’s original analysis was conducted. Second, we assess individual differences for the remember/know paradigm and compare them to individual differences in the sure/unsure paradigm from Experiment 3.

### Original analysis

In the section ”Statistical models for data analysis”, we describe the original analyses conducted by Gardiner and Java (1990): Two 2 × 2 ANOVA analyses, one for *remember* and *know* response frequencies for old items, and one for *remember* and *know* response frequencies for lures. For our main analyses, we did not use the same approach, as it is not well suited for the data at hand. Yet, in order to allow for a direct comparison between the results reported by Gardiner and Java and our analyses, we provide the ANOVA results here. As a reminder, for old items, Gardiner and Java found no significant main effects for response type (*remember* vs. *know*) or item type (word vs. nonword), but they found a significant interaction between the two factors. For lures, Gardiner and Java found a significant main effect of response type but no significant main effect of item type nor a significant interaction.

Tables 4 and 5 show the ANOVA results for Experiments 1, 2, and 4 for old items and lures, respectively. For old items, the only significant effect is the main effect of response type in Experiment 4. In neither of the experiments could we find a significant interaction effect. For new items, the pattern is a bit more mixed across experiments. In Experiment 1, none of the tests are significant. For Experiment 2, all of them are at an *α* = 0.05 level. One reason might be the increased accuracy for new nonwords in Experiment 2 leading to less *know* responses for nonwords. The effect sizes are small, however, expect for the main effect of response type. For Experiment 4, there are small but significant main effects at an *α* = 0.05 level, but the interaction is not significant.

**Table 4:** Original ANOVA analysis for old items

Effect	*F*	$\mathit {df}_{1}^{GG}$	$\mathit {df}_{2}^{GG}$	*MSE*	*p*	$\hat {\eta }^{2}_{G}$
Experiment 1						
Item: Word vs. nonword	0.23	1	51	8.33	.633	.000
Response: Remember vs. know	2.47	1	51	84.35	.123	.032
Item × Response	0.64	1	51	20.39	.428	.002
Experiment 2						
Item: Word vs. nonword	0.04	1	50	5.36	.833	.000
Response: Remember vs. know	0.20	1	50	54.13	.657	.002
Item × Response	0.58	1	50	25.49	.449	.003
Experiment 4						
Item: Word vs. nonword	1.85	1	49	6.47	.180	.002
Response: Remember vs. know	10.46	1	49	48.62	.002	.077
Item × Response	2.08	1	49	50.59	.156	.017

**Table 5:** Original ANOVA analysis for lures

Effect	*F*	$\mathit {df}_{1}^{GG}$	$\mathit {df}_{2}^{GG}$	*MSE*	*p*	$\hat {\eta }^{2}_{G}$
Experiment 1						
Item: Word vs. nonword	1.38	1	51	8.38	.246	.003
Response: Remember vs. know	1.59	1	51	34.52	.212	.015
Item × Response	0.55	1	51	13.38	.463	.002
Experiment 2						
Item: Word vs. nonword	12.29	1	50	5.55	.001	.038
Response: Remember vs. know	42.62	1	50	11.20	< .001	.218
Item × Response	6.20	1	50	6.41	.016	.023
Experiment 4						
Item: Word vs. nonword	4.37	1	49	4.83	.042	.008
Response: Remember vs. know	5.59	1	49	24.94	.022	.050
Item × Response	0.71	1	49	9.65	.404	.003

In summary, we consistently find no significant interaction for old items, and we consistently find an effect of response type for new items. Both of these results are more in line with single-process than dual-process accounts. Across experiments, we do not find a consistent effect of item type. We suspect that this effect is weak at best.

### Remember–know vs. sure-unsure

In light of our results, we may speculate about the role of remember–know instructions compared to more conventional confidence-rating instructions. The confidence-rating experiment, Experiment 3, revealed a strong, consistent preference for the *sure* response relative to the *unsure* response with little individual differences. People are sure about what they know and they are clearly indicating so. As a result, the standard deviations for *unsure* response proportions are relatively low with 0.123 for nonwords and 0.122 for words. This preference can be contrasted with the response pattern for *remember* and *know* from Experiments 1, 2, and 4. Here, we see a lack of preference as well as more variability across individuals. The pattern of individual response proportions is extreme: Some individuals almost exclusively respond *remember* to old items while others almost exclusively respond *know* to old items. The standard deviations for *know* responses are therefore somewhat higher. For example, the standard deviations for nonwords and words in Experiment 2 are 0.206 and 0.161, respectively.

We speculate that participants have a vague idea at best what *remember* and *know* mean, and the vagueness leads to arbitrary, subjective decisions about their memory that are not indicative of underlying processes (Naveh-Benjamin & Kilb, 2012), and that may even be affected by perceptual information (Mulligan, Besken, & Peterson, 2010). These vague subjective definitions of remember–know can be contrasted with the treatment of the sure/unsure distinction where participants are more consistent and more sure of their memory. Admittedly, the instructions of the remember–know task used here could be improved by, for example, providing practice trials where reasons for the responses have to be provided and feedback is given. Yet, the majority of remember–know instructions in the literature are verbal. In Experiment 4, we even used instructions that held up in previous comparison (Geraci et al., 2009). It may therefore be reasonable to assume that participants had similar difficulties in other R/K paradigms as they had in our studies, at least the ones that used the identical instructions as our Experiment 4. Another potential improvement to the RKN-task used by Gardiner and Java (1990), and therefore us, is the usage of a *guess* response option a is common in more recent RK tasks. Such a *guess* option might clean up the pattern to a degree by reducing the number of *know* responses and possibly the number of false alarms. However, Migo et al., (2012) noted that adding an additional response option might complicate the task even more. In light of our results, this potential issue is a valid concern.

## General discussion

In this paper, we sought to replicate Gardiner and Java (1990). We consider their Experiment 2 to be the strongest direct behavioral evidence for the distinct memory processes of conscious recollection and automatic activation. Across two labs, the critical data patterns—a crossover interaction for old items and an invariance for new items—could not be found. Instead, there is seemingly no effect or a small effect of lexical status. Moreover, Bayesian model comparison of all four experiments supports the simpler single-process model over the more complicated dual-process alternative.

### Procedural and analytic differences

There are several procedural and analytic differences between our experiments and Gardiner and Java’s. We think our choices are improvements that rectify limitations in the original design. Here is a review of the major differences:

1. Increased sample sizes: Gardiner and Java ran experiments with 20 participants observing 60 test items for a total of 1200 observations. We ran experiments with at least 50 participants observing at least 100 test items for a total of at least 5000 observations. Hence, our experiments afford greater resolution to see effects and invariances.
2. Decreased retention interval: Our retention interval was 10 min rather than 24 h. During this retention interval, all participants performed the same intervening task. This shorter retention period allowed us to increase the number of items at study and test while maintaining a reasonable level of overall performance. Moreover, we could ensure that participants were having the same experience in the retention interval. Importantly, Gardiner and Java did not consider the long retention interval essential, and note it was used only to avoid ceiling effects which we avoid with more items. Additionally, previous attempted replications of Gardiner and Java’s Experiment 2 did not use the 24-h retention interval (Geraci et al., 2009; Rajaram et al., 2002).
3. Computerized, sequential presentation: Gardiner and Java used hand-written items on cards and paper. We computerized the task. In doing so, we used a sequential presentation at test. This method contrasts favorably with Gardiner and Java’s simultaneous presentation at test, in which all test items were presented on a single piece of paper. Our approach is much more in line with the procedure employed nowadays by most recognition memory researchers, and the sequential nature reduces response dependencies across items. Additionally, the paper-method appears to have introduced a response bias in Gardiner and Java’s procedure where participants preferred *new* responses (i.e., *not* circling an item) over *old* responses (i.e., circling an item). We eliminate this bias.
4. Analysis through model comparison: Gardiner and Java used separate ANOVAs to analyze their data, and analyzed response proportion as a function of response option (remember vs. know) and lexicality. Unfortunately, ANOVA is grossly inappropriate in this application. We take a more appropriate and sophisticated approach by instantiating different theoretical positions as formal statistical models and then use Bayesian model comparison to draw inferences. This approach of using custom-tailored, theoretically specific linear models to answer critical questions should be attractive across cognitive psychology, and we refer interested readers to Haaf et al., (2019), Rouder et al., (2016), and Rouder, Haaf, and Aust 2018. Notably, even when we apply the original analysis to our experiments we cannot replicate the original findings.

In summary, although our experiments differ in a few aspects from Gardiner and Java, we feel that our choices provide clear improvements. We thought carefully and deliberately about each, understood why we were making the change, and documented each in the preregistration documents (https://osf.io/873sg/, https://osf.io/k2ve3/, and https://osf.io/92ng3).

### Signal or noise?

The remaining question is why our results differ from Gardiner and Java’s. Some readers, especially those predisposed to the dual-process account, may remain unsure whether our failure to replicate reflects procedural changes. We suspect most readers will not object to computer presentation, appropriate analysis, or increased sample size. Some may wonder about the effect of the 10-min vs. 24-h retention period or the effect of sequential vs. simultaneous testing. We note that there is no theoretical reason to think that dual-process signatures would be observable *only* after a day or *only* with simultaneous tests. In fact, it stretches common sense that such a fundamental mnemonic signature, if it existed, would be observable in such an unanticipated, limited set of conditions. Moreover, if these conditions are needed to observe the critical dual-process pattern, then the vast majority of remember–know experiments in the literature are fatally flawed.

It is more likely that Gardiner and Java have misinterpreted noise for signal. Their studies were relatively underpowered and their analysis is characterized by high true type I error rates in interaction contrasts from naturally occurring negative correlation across response options. When we correct these flaws, we see no signature of two processes.

### Conclusions

Here we have reported a failure to replicate an important behavioral finding with the remember/know task. It is important to note that this is not the only source of behavioral evidence leveraged by dual-process theorists in favor of distinct recollection and familiarity processes. As noted in the introduction, the shape or ROC curves (Yonelinas, 1999), double-dissociations (Schacter and Tulving, 1994), and speed effects (Besson et al., 2012; McElree et al., 1999) have all been cited in support of the idea that two processes are responsible for recognition memory. However, in these cases, there are corresponding demonstrations of doubt showing that these findings are often consistent with the simpler single-process view (e.g., Dougal and Rotello 2007; Dunn 2004; Mulligan and Hirshman 1995; Osth, Dunn, Heathcote, & Ratcliff 2019; Province and Rouder 2012.

For the current study, we focus on the remember/know findings of Gardiner and Java (1990) as they distinctly support two processes and rule out alternative single-process explanations. Of note is that these results are immediately interpretable without the need to fit formal process models. Developing more constrained models and focused tests will be important for distinguishing single- and dual-process accounts of recognition memory. In addition, we believe examining the replicability of key findings in the recognition memory literature will be equally important.

## Open Practices Statement

The authors advocate for and adhere to a fully transparent research pipeline (Rouder, Haaf, & Snyder, 2019). This transparency includes preregistration of all four experiments, open data, and open analysis code.

- Preregistration of Experiment 1 can be found here: https://osf.io/873sg/; Experiments 2 and 3 are preregistered at https://osf.io/k2ve3/; Experiment 4 is preregistered at https://osf.io/92ng3.^2^
- Data for Experiments 1 and 3 were *born open* (Rouder, 2016), that is, they were uploaded to a public repository nightly during data collection, and are available https://github.com/PerceptionCognitionLab/data1/tree/master/repGardinerJava/RKN_replication/RKN_exp1 and https://github.com/PerceptionCognitionLab/data1/tree/master/repGardinerJava/exp2/RKN_replication/RKN_exp2/SU.
- Data from Experiments 2 and 4 were made public after data collection and are available https://github.com/PerceptionCognitionLab/data0/tree/master/rm-gardiner-java.
- The document for this paper, with all text and code, can be found at https://github.com/PerceptionAndCognitionLab/rm-gardiner-java/tree/public/papers/current.

Please contact the first author in case there are any questions about the data or analysis.

## Appendix A: Material

Below are the words and nonwords presented to the participants during the study phase. The items in italic are the ones only used for Experiment 1. The other items are used for Experiments 1, 2, and 3.

*Words*

“BATH”, “BEEF”, “BIRD”, “BLUE”, “BOOK”, “CAKE”, *“CALL”*, “CASH”, “COAT”, “COLD”, “DATE”, “DOOR”, “FACE”, “FACT”, “FEET”, “GATE”, “GIRL”, “GOOD”, “HALF”, “HALL”, “HAND”, *“HAVE”*, “HEAD”, *“HELP”*, *“HOLD”*, “HOME”, “KISS”, “KNEE”, “LEFT”, “LIFE”, “LIKE”, “LINE”, “LOOK”, “MAKE”, “MIND”, “NOTE”, “PAGE”, “RAIN”, “REST”, “ROAD”, *“ROOM”*, “SALT”, “SEAT”, *“SELF”*, “SHOP”, “SKIN”, “SNOW”, *“SOAP”*, *“SOFT”*, “SONG”, “TALK”, “TIME”, “TREE”, “WALK”, *“WANT”*, *“WARM”*, “WASH”, “WIND”, “WORK”, “YEAR”

*Nonwords*

“WUIL”, “RILM”, “DENC”, *“ZYSE”*, “LODD”, “CHIE”, “SEFS”, *“JAUK”*, “GWIC”, “WONE”, “PLOK”, “DAPT”, “RETE”, “KLIB”, “SIME”, “LATT”, “SWAZ”, “DUFE”, “WONS”, *“HEWF”*, “MENC”, *“ZUNK”*, “COLV”, “CLOF”, “ABST”, *“YOGG”*, “DAUV”, “VEUL”, “HOAB”, “DOYS”, “SPIZ”, “NARN”, *“ZELF”*, “YAIL”, “CWEB”, “NOGE”, *“WONC”*, “DWEK”, “ZARC”, *“GWUZ”*, *“NALN”*, “HESP”, “JALT”, “UFTS”, “CWUL”, “KEPH”, “MYDE”, “SOTE”, “CHUR”, “FOMB”, “FOSK”, “TRUV’, ’“SNUZ”, “TASP”, “NAUC”, “VABB”, “ZEAM”, *“TUCE”*, “JOSP”, “LORT”

In Experiment 4, we used stimuli provided by Rajaram et al., (2002):

*Words*

“BEAN”, “LIMB”, “GATE”, “FOAM”, “WASH”, “BOMB”, “HANG”, “CARD”, “BEND”, “JUMP”, “FARE”, “BAIL”, “SALT”, “BOND”, “HAIR”, “MEET”, “PAIN”, “KING”, “TUNE”, “BITE”, “COAT”, “IRON”, “DRIP”, “FERN”, “DATE”, “RACE”, “HOME”, “PART”, “YEAR”, “COME”, “SINK”, “WORN”, “HALL”, “HEAR”, “DESK”, “CELL”, “BACK”, “MUCH”, “SEAT”, “DEAR”, “COOK”, “SOLD”, “MALE”, “FOOL”, “SAFE”, “PALE”, “GAME”, “TEST”, “BIRD”, “MAID”, “BOAT”, “HILL”, “LOAF”, “DOVE”, “LEAF”, “SILK”, “DUST”, “SONG”, “WALL”, “FINE”

*Non-Words*

“ABST”, “IGST”, “ORTT”, “AFTH”, “FARB”, “HIRP”, “KLIB”, “SLIG”, “TADE”, “PATE”, “INPS”, “ORKS”, “BLOS”, “TRAS”, “JOSP”, “CADT”, “AELT”, “OURT”, “SOTE”, “PIGE”, “DOOT”, “GEEL”, “HIPT”, “PIFT”, “GLAF”, “JASL”, “FILT”, “NIST”, “KNOO”, “SLEE”, “FLOU”, “SPOA”, “GORT”, “BOPT”, “NOST”, “LOBT”, “DELP”, “NOPH”, “GINP”, “DOPT”, “BILP”, “FILK”, “ILST”, “OLND”, “NORT”, “FOLT”, “LOPT”, “NULB”, “CHUR”, “TROB”, “EGST”, “TOLR”, “AHLL”, “OBLL”, “SELB”, “TILB”, “LORT”, “LONT”, “INPT”, “ONLT”

## Appendix B: Instructions

### Study Instructions

The same study instructions were used for Experiments 1, 2, and 3: In this experiment, you will be presented with strings of four letters to remember. Sometimes these letters will make a word (for example, CAPE), or sometimes they will be a ’non-word’, which is word-like but has no meaning (for example, LARC). Each item (word or non-word) will be presented one at a time in the middle of the screen. Pay close attention to each and try to remember them. Once you have studied all of the items, you will be given another task to do for 10 min. After that, you will be given a recognition test. Press SPACE to begin.

### Test instructions for Experiment 1

The following instructions were given on the screen: Now is the memory test for the words and nonwords you studied before. You will see a single item at a time; some of these will be from the set you studied in the first part of the experiment (OLD), others will be ones you did not study (NEW). Please work carefully through each item, indicating for each one whether you recognize it from the first part of the study or not. If you recognize an item, please click the OLD button. If you do not recognize it, please click the NEW button. Additionally, as you make your decision about recognizing each word/ nonword, bear in mind the following: Often, when remembering a previous event or occurrence, we consciously RECOLLECT and become aware of aspects of the previous experience. At other times, we simply KNOW that something has occurred before, but without being able consciously to recollect anything about its occurrence or what we experienced at the time. Thus, in addition to your indicating your recognition of a word/ nonword from the original study set, you will be asked to click “R” to show that you recollect the item consciously, or click “K” if you feel you simply know that the item was in the previous study set. So, for each item that you recognize as OLD, please click “R” if you recollect its occurrence, or “K” if you simply know that it was shown in the first part of the experiment.

Afterwards, the experimenter provided a few every-day examples of remembering and knowing. These examples were neither standardized nor recorded.

### Test instructions for Experiment 2

The following instructions were given on the screen: After you decide an item is old, we would like you to tell us how you know that. We are going to give you two choices. One is what we call recollection. To recollect something means you remember seeing it. Perhaps you remember a specific thought or perhaps you remember what came before or after. The key here is that you remember some details about the experience of studying that item. Another way of that you may think an item is old is to know it. Knowing means that you know its old, but can’t recall any of the details. But you still know that item was studied. If you are recollecting an item, please hit the “R” button. If you know it is old, hit the “K” button.

Afterwards, the experimenter gave further verbal instructions following a script: OK, so let’s do a few examples. Suppose you are asked about the word FROG, and you happen to remember seeing frog because you thought about Kermit. In this case, you are recollecting and should press “R”. Recollection is when you can remember actually seeing the word. But suppose, alternatively, in your gut, you know FROG was there, but can’t actually remember seeing the word at study. In this case, press “K”. The difference between recollection and knowing is kind of like trying to figure out where you parked your car at the mall. Sometimes you can recall the act of parking including a detail or two like the car next to you or the song on the radio. Other times you just walk back there because you know where to go.

### Test instructions for Experiment 3

The following instructions were given on the screen: After you decide an item is old, we would like you to tell us how sure you are in your decision. If you are very sure it is old, that is you might even bet a lot of money on it, hit the “S” button for sure. If you are not quite this sure, that is, you wouldn’t want to bet on it, hit the “U” button for unsure.

These instructions were supported by the following verbal instructions: OK, so let’s do a few examples. Suppose you are asked about the word FROG, and you happen to strongly remember seeing FROG, and you are equally sure it wasn’t TOAD or anything like that. Hit “S” for sure, bet on it. But suppose your memory is a bit fuzzier. Maybe there was reptile, maybe toad, maybe not. Then hit “U” for unsure. Don’t bet on things you don’t know for sure.

### Read instructions for Experiment 4

This is a memory test. On the screen you will see one item at a time. By ’item’ we mean either a word or a non-word. Please indicate whether you recognize each item as having been presented earlier during the study session. If you recognize the item from the study list you should click the OLD button. If you do not recognize the item you should click the NEW button. The computer will wait for you to decide whether or not you studied that item earlier. If you recognize the item, then please judge whether you Remember the item from the list or you know it was there. The following descriptions will help you make the distinction between these two post-memory judgments. You should make a remember judgment if you can consciously recollect its prior occurrence. Remember is the ability to become consciously aware again of some aspect or aspects of what happened or what was experienced at the time the word was presented (e.g., aspects of the physical appearance of the item, or of something that happened in the room, or of what you were thinking or doing at the time). In other words, the ‘remembered’ item should bring back to mind a particular association, image, or something more personal from the time of study, or something about its appearance or position (i.e., what came before or after that item). You should make a know judgment if you recognize the item from the study list, but you cannot consciously recollect anything about its actual occurrence or what happened or what was experienced at the time of its occurrence. In other words, respond ‘know’ when you are certain that you recognize the item, but it fails to evoke any specific conscious recollection from the study list. To further clarify the difference between these two judgments (remembering and knowing), here are a few examples. If someone asks for you name, you would typically respond in the ‘know’ sense, without becoming consciously aware of anything about a particular event or experience. However, when asked the last movie you saw, you would typically respond in the ‘remember’ sense, that is, becoming consciously aware again of some aspects of the experience of seeing the movie. Now, I will ask you to describe instances from your own life that you would classify as Remember responses and Know responses to make sure that the distinction between these two states is clear. (At this point stop and ask subjects for examples using both categories.) To reiterate, you will see a list of items and you will judge whether you recognize the words as having been presented earlier. If you recognize the word, click the OLD button then you will try to indicate how you recognize the particular word, by clicking the ‘R’ button for ‘remember’ or the ‘K’ button for ‘know’. Importantly, if you indicate that you do not recognize the word, then you will simply move on to the next item since it will not be relevant (you can’t remember a word that you said you did not study!). Please think carefully about each item and try not to guess. When you are ready you may begin.

## Appendix C: Analysis Code

This paper was written in R-Markdown. In R-Markdown, the text and the code for analysis may be included in a single document. The document for this paper, with all text and code, can be found at https://github.com/PerceptionAndCognitionLab/rm-gardiner-java/tree/public/papers/current. We used R (Version 4.0.2, R Core Team (2017)) and the R-packages *BayesFactor* (Version 0.9.12.4.2, Morey and Rouder (2015)), *coda* Version 0.19.3, Plummer, Best, Cowles, & Vines, (2006), *knitr* (Version 1.29, Xie (2015)), *Matrix* (Version 1.2.18, Bates and Maechler (2016)), *papaja* (Version 0.1.0.9997, Aust and Barth (2017)), *plyr* (Version 1.8.6, Wickham (2011)), *reshape2* (Version 1.4.4, Wickham (2007)), *rvest* (Version 0.3.5, Wickham (2016)), *stringr* (Version 1.4.0, Wickham (2017)), and *xml2* (Version 1.3.2, Wickham et al., (2017)) for all our analyses.

## Footnotes

1 Preregistration of Experiment 1 can be found here: https://osf.io/873sg/; Experiments 2 and 3 are preregistered at https://osf.io/k2ve3/; and Experiment 4 was preregistered at https://osf.io/92ng3.

2 Preregistration of Experiment 4 was written in December 2019 but unfortunately only uploaded in April 2020. No changes were made in the meantime, and the preregistration was uploaded prior to data analysis.
